# Supplementary material for: Regional anaesthesia practice in public hospitals in Botswana: A cross-sectional study
Source: PLoS One. 2023 Dec 19;18(12):e0295932. doi: 10.1371/journal.pone.0295932 (PMC10729981; doi:10.1371/journal.pone.0295932)
Supplement: Attachment — Submitted filename: Reviewers_Responses letter_28 Oct.docx [file pone.0295932.s002.docx]

Eugene TUYISHIME, MBBS, MSc, 28^th^ October, 2023

Anesthesiologist

Assistant Program Director,

Anesthesia and Critical Care department

University of Botswana,

Phone: + 26774504984

Email: [tuyishime36@gmail.com](mailto:tuyishime36@gmail.com)

To: Editor of Plos One Journal

Dear Editor, Silvia Fiorelli,

RE: Re-submission of the manuscript/PONE-D-23-09813 entitled “**Regional anaesthesia practice in public hospitals in Botswana: A cross-sectional study**”

I am pleased to submit a revised version of our study to Plos One journal.

Our responses to your comments and the reviewer’s questions are highlighted in text in blue both in this letter and the manuscript text.

Thank you for your consideration.

Sincerely,

Dr Eugene Tuyishime

**Editor and Reviewer Comments:**

**Academic Editor** (Silvia Fiorelli)

Journal Requirements:

When submitting your revision, we need you to address these additional requirements.

1. Please ensure that your manuscript meets PLOS ONE's style requirements, including those for file naming. The PLOS ONE style templates can be found at
   <https://journals.plos.org/plosone/s/file?id=wjVg/PLOSOne_formatting_sample_main_body.pdf> and 
   <https://journals.plos.org/plosone/s/file?id=ba62/PLOSOne_formatting_sample_title_authors_affiliations.pdf>

We have addressed the style requirements as suggested.

**Reviewer #1:**

Sorry for the inconveniences and delay in the publication process.
Despite limitations on methodological robustness the authors tried their best to incorporate the given feedback, the research work is imperative, informative and gives insight for policymakers, funding agencies and professional development.

Hence, I suggest the acceptance of the manuscript with minor revisions.

Thank you for your feedback and suggestions throughout the review process.

**Reviewer #2:**

Dear authors,

Here you receive my 2nd review regarding the manuscript with the title: Regional anaesthesia practice in public hospitals in Botswana: A cross-sectional study (Regional anaesthesia practice in public hospitals in Botswana) and Manuscript Number: PONE-D-23-09813R1.

The authors have carefully edited the manuscript and taken our previous criticisms seriously to improve understanding and clarity of the article and have answered our questions accordingly.

I only have one text comment. Please look at the Plos One’s Style requirements more carefully and accurately as requested by the Academic Editor (Silvia Fiorelli).

There should be uniformity in the way references are numbered in the text, ie with or without a space after the last letter of a word, with or without brackets, or as a superscript.

See examples on the following pages:
page 5: … guidelines(17). And more examples on this page and further on this page and in the manuscript
page 6: … hospital(15,21) and line 4: …training 15 and in the discussion on P13 : … women 3-5
and page 14: .. program (3-6) or …LRS (3-6, 12,13)
Please check other places within the article also?

Thank you for your feedback. We have addressed the style requirements throughout the manuscript as suggested.
